# High-Altitude Adaptation: Mechanistic Insights from Integrated Genomics and Physiology

**DOI:** 10.1093/molbev/msab064

**Published:** 2021-03-04

**Authors:** Jay F. Storz

**Affiliations:** School of Biological Sciences, University of Nebraska, Lincoln, NE, USA

**Keywords:** adaptation, altitude, hypoxia, genotype–phenotype association, hypoxia-inducible factor, *EPAS1*

## Abstract

Population genomic analyses of high-altitude humans and other vertebrates have identified numerous candidate genes for hypoxia adaptation, and the physiological pathways implicated by such analyses suggest testable hypotheses about underlying mechanisms. Studies of highland natives that integrate genomic data with experimental measures of physiological performance capacities and subordinate traits are revealing associations between genotypes (e.g., hypoxia-inducible factor gene variants) and hypoxia-responsive phenotypes. The subsequent search for causal mechanisms is complicated by the fact that observed genotypic associations with hypoxia-induced phenotypes may reflect second-order consequences of selection-mediated changes in other (unmeasured) traits that are coupled with the focal trait via feedback regulation. Manipulative experiments to decipher circuits of feedback control and patterns of phenotypic integration can help identify causal relationships that underlie observed genotype–phenotype associations. Such experiments are critical for correct inferences about phenotypic targets of selection and mechanisms of adaptation.

## Introduction

Genome-wide scans of DNA polymorphism in high-altitude human populations have revealed numerous loci that exhibit signatures of positive selection, many of which represent plausible candidate genes for hypoxia adaptation (Bigham et al. 2009, 2010; Aggarwal et al. 2010; Beall et al. 2010; Simonson et al. 2010; Yi et al. 2010; Peng et al. 2011; Xu et al. 2011; Alkorta-Aranburu et al. 2012; Scheinfeldt et al. 2012; Huerta-Sanchez et al. 2013; Xiang et al. 2013; Xing et al. 2013; Crawford et al. 2017; Hu et al. 2017; Yang et al. 2017; Arciero et al. 2018; Jeong et al. 2018). In Tibetan highlanders, such genome scans have consistently implicated central components of the hypoxia-inducible factor (HIF) signaling pathway, which orchestrates the transcriptional response to hypoxia (Beall et al. 2010; Simonson et al. 2010; Yi et al. 2010; Peng et al. 2011, 2017; Xu et al. 2011; Xiang et al. 2013; Yang et al. 2017; Jeong et al. 2018). Members of the HIF family of transcription factors exert O_2_-dependent control over the tissue-specific expression of myriad target genes and regulate diverse facets of the physiological response to hypoxia, including respiration, blood flow, vascular remodeling, and intermediary metabolism (Kaelin and Ratcliffe 2008; Lendahl et al. 2009; Majmundar et al. 2010; Greer et al. 2012; Semenza 2012, 2014; Samanta et al. 2017). Some HIF pathway genes such as *EPAS1* (*endothelial PAS domain containing protein 1*), which encodes the O_2_-sensitive α subunit of the HIF-2 transcription factor, exhibit statistical evidence for positive selection in multiple high-altitude populations and species (Witt and Huerta-Sanchez 2019; Storz and Cheviron 2021). At face value, such patterns would seem to suggest that similar adaptive solutions have evolved repeatedly in response to the shared physiological challenge of environmental hypoxia. However, the extent to which shared signatures of positive selection reflect similarities in causal paths and phenotypic outcomes remains an open question. Comparative studies of systemic physiology in high-altitude humans and other vertebrates have revealed a far greater diversity of adaptive mechanisms than might be suggested by cross-referencing lists of candidate genes and gene ontology categories (Monge and Leon-Velarde 1991; Hochachka 1998; Beall 2006, 2007; Storz, Scott, et al. 2010; Gilbert-Kawai et al. 2014; Petousi and Robbins 2014; Ivy and Scott 2015; McClelland and Scott 2019; Storz et al. 2019; Storz and Scott 2019; O’Brien et al. 2020). 

In studies of environmental adaptation, documenting an association between genotype and phenotype represents a necessary first step that can guide the design of follow-up experiments to test hypotheses about causal mechanisms. To identify and characterize mechanisms of adaptation to high-altitude hypoxia, focal phenotypes ideally represent fitness-related measures of whole-organism performance that reflect integrated physiological capacities (Bennett 1991; Storz et al. 2015, 2019; McClelland and Scott 2019; Storz and Scott 2019). Integrating such measures of systemic physiology with analyses of subordinate traits (respiratory, cardiovascular, and metabolic) can provide mechanistic insights into the chain of causation linking genotype and selected phenotype. For example, common-garden experiments involving high- and low-altitude deer mice (*Peromyscus maniculatus*) revealed that highland natives have evolved enhanced aerobic performance capacities in hypoxia owing to derived changes in numerous subordinate traits that alter the flux capacity of the O_2_-transport system, the oxidative capacity of tissue mitochondria, and the relationship between O_2_ consumption and ATP synthesis (Cheviron et al. 2012; Cheviron, Connaty et al. 2014; Lui et al. 2015; Scott et al. 2015, 2018; Ivy and Scott 2017; 2018; Lau et al. 2017; Mahalingam et al. 2017, 2020; Tate et al. 2017, 2020; Dawson et al. 2018; Nikel et al. 2018; Storz et al. 2019; Ivy et al. 2020). In addition to the examination of physiological performance capacities, the challenges of mammalian pregnancy at high altitude suggest that direct measurements of female reproductive success may capture significant variation in the fertility component of fitness (Moore 2001; Browne et al. 2015; Niermeyer et al. 2015; Grant et al. 2020).

Below I review and synthesize results from recent studies that illustrate how genomic data can be integrated with experimental physiology to yield insights into mechanisms of high-altitude adaptation. The review of recent work is organized according to the following progression, which does not necessarily follow the exact chronological sequence in which the various studies were performed: 1) analysis of genome-wide polymorphism data yields the discovery of candidate genes for hypoxia adaptation based on statistical signatures of positive selection; 2) guided by prior knowledge about pathways affected by allelic variation in a given candidate gene, experimental measurements of relevant physiological traits reveal altitude-related differences in mean phenotype; 3) experiments involving subjects with known genotypes reveal an association between specific allelic variants and phenotype; and 4) manipulative experiments using reverse genetics yield insights into causal mechanisms that underlie evolved differences in phenotype between high- and low-altitude natives. To date, several studies have followed this progression (with varying levels of completeness) through 2 or 3 of these steps. Such studies demonstrate how indirect, retrospective inferences about selection based on population genomic analyses can be integrated with mechanistic experiments to yield discoveries about the functional biology of adaptation. In genomic studies that involve relatively sparse or superficial measurements of physiological traits, a key interpretative challenge is that observed genotypic associations with hypoxia-responsive phenotypes may not reflect direct, causal relationships. Instead, such associations may reflect indirect effects of selection-mediated change in a separate, unmeasured trait (e.g., an upstream step in the same pathway) that is coupled with the measured trait via feedback regulation.

## Fitness-Related Variation in Aerobic Performance Capacity in Hypoxia

In endotherms living in cold, hypoxic conditions at high altitude, ecologically important measures of whole-organism performance, such as capacities for sustained exercise and thermogenesis, are directly related to aerobic metabolism. An animal’s rate of aerobic metabolism can be measured as the rate of O_2_ consumption (*V*O_2_, typically measured in units of ml·min^−1^·kg^−1^) because O_2_ is required for ATP synthesis via oxidative phosphorylation in the mitochondria. The maximal rate of aerobic metabolism, which is measured by the maximal rate of O_2_ consumption (*V*O_2max_), is an index of whole-organism aerobic performance that reflects the integrated functioning of the cardiopulmonary/cardiovascular systems and muscle metabolism (Weibel et al. 1991; Taylor et al. 1996). This integrated functioning determines the flux capacity of the O_2_ transport pathway, which consists of serially integrated physiological processes representing diffusive and convective transfer steps (ventilation, pulmonary O_2_ diffusion, circulatory O_2_ delivery, and tissue O_2_ diffusion) that culminate in mitochondrial O_2_ utilization. *V*O_2max_ can be elicited by challenging an animal’s exercise capacity (e.g., via forced treadmill running) or by challenging thermoregulatory capacity via acute cold exposure. When exposed to extreme cold, most eutherian mammals increase metabolic heat production via shivering or non-shivering thermogenesis. Thus, progressive reduction of ambient temperature will eventually elicit an animal’s maximal rate of metabolic heat production when it reaches the upper limit of O_2_ consumption.

At high altitude, the reduced partial pressure of O_2_ (*P*O_2_) of inspired air imposes constraints on aerobic metabolism and therefore impairs capacities for sustained exercise and thermogenesis (West 2006; Brutsaert 2008; Gonzalez and Kuwahira 2018; McClelland and Scott 2019). For endothermic vertebrates living at high altitude, the combination of increased thermoregulatory demand (due to low temperature) and reduced capacity for aerobic thermogenesis (due to low *P*O_2_) suggests that variation in cold-induced *V*O_2_max may have especially important fitness consequences. In small endotherms like rodents, the capacity for sustained thermogenesis in hypoxia may be critical for survival during daily or seasonal periods of extreme cold. In such conditions, individuals with higher capacities for aerobic thermogenesis can maintain their body core temperature at lower ambient air temperatures. Such individuals are less likely to succumb to hypothermia and are able to expand the active period of the torpor cycle, thereby increasing opportunities for foraging, mating, and other activities that may contribute to lifetime reproductive success (Hayes and O’Connor 1999; Sears et al. 2006). Measurements of field metabolic rates of high-altitude deer mice revealed that these animals are often operating close to their aerobic performance limits (Hayes 1989a, 1989b) and survivorship studies confirm that thermogenic capacity is subject to strong directional selection under natural conditions (Hayes and O’Connor 1999).

In summary, hypoxic *V*O_2_max provides an ecologically relevant and physiologically integrative measure of whole-organism aerobic performance. Quantification of the decrement in *V*O_2_max with increasing hypoxia can therefore provide the basis for an operational measure of high-altitude adaptation (Brutsaert 2008, 2016; McClelland and Scott 2019; Storz et al. 2019).

### Association of *EGLN1* Variants with Aerobic Capacity in Hypoxia

Quechua have lived for millennia in high-altitude regions of the Andes in Peru and Bolivia (Rademaker et al. 2014), and multiple lines of evidence indicate that they have higher limits of work performance in hypoxia relative to their nonindigenous and mestizo compatriots (Hochachka et al. 1991; Frisancho et al. 1995; Brutsaert et al. 2003; Brutsaert 2008, 2016). In fact, the impressive physical work capacities of Andean natives at high altitude were chronicled by Spanish conquistadors nearly 500 years ago (Monge 1948). Andean natives maintain a higher mean *V*O_2_max in hypoxia and suffer a smaller decrement in *V*O_2_max with increasing hypoxia relative to nonnative residents at the same altitude (Frisancho et al. 1995; Brutsaert et al. 2003; Brutsaert 2008, 2016). The genetic basis of this enhanced performance in hypoxia has yet to be elucidated. However, results of genome scans in native Andeans suggest that variation in HIF genes such as *EGLN1* (*egl-9 family hypoxia inducible factor*) may have contributed to adaptation (Bigham et al. 2009, 2010; Foll et al. 2014; Crawford et al. 2017) and it is possible that some fraction of the phenotypic response to past selection is captured by general performance measures such as *V*O_2_max. Accordingly, Brutsaert et al. (2019) used a sample of 523 subjects (Quechua highlanders and non-Hispanic lowlanders) to test for an association between noncoding *EGLN1* SNP variants and *V*O_2_max in hypoxia. *EGLN1* encodes a prolyl hydroxylase (PHD2) that induces degradation of HIF in an O_2_-dependent manner. The choice of *EGLN1* as a focal gene for the association study was also motivated by experimental evidence that loss-of-function mutations in *EGLN1* are associated with a misregulation of O_2_ homeostasis, which affects erythropoiesis in humans (Percy et al. 2006; Lee and Percy 2011) and erthropoietic and ventilatory responses to environmental hypoxia in mice (Arsenault et al. 2013; Bishop et al. 2013).

**Fig. 1.**
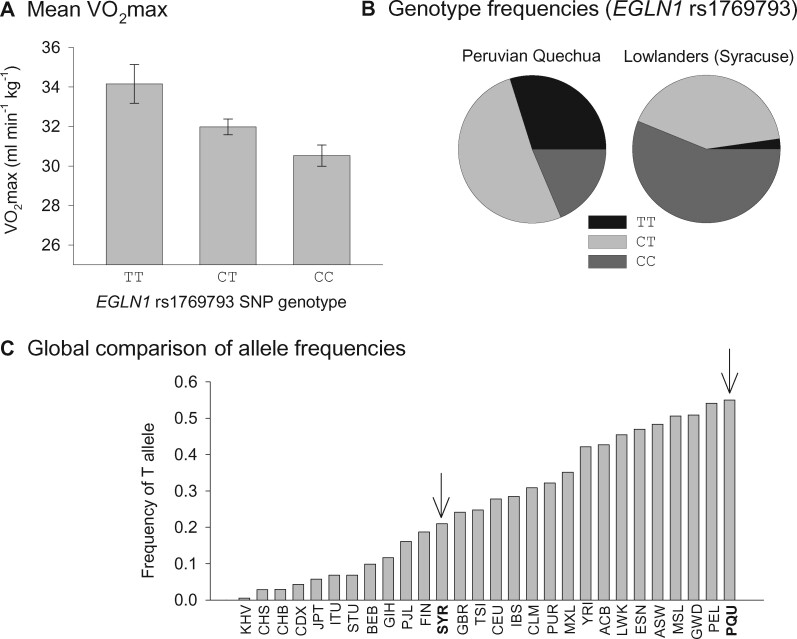
In high-altitude Quechua, noncoding SNPs in *EGLN1* are associated with aerobic exercise capacity (*V*O_2_max) in hypoxia. (*A*) Marginal mean values of *V*O_2_max for three alternative *EGLN1* SNP genotypes (error bars = SEM) in a sample of Peruvian Quechua highlanders and non-Hispanic lowlanders. (*B*) Genotype frequencies for *EGLN1* rs1769793 in Peruvian Quechua and non-Hispanic lowlanders from Syracuse, NY. The “TT” genotype is associated with high *V*O_2_max in hypoxia. (*C*) Allele frequencies of the T allele from the 1000 Genomes Project. Arrows denote frequencies for the Peruvian Quechua (PQU) and non-Hispanic Syracuse (SYR) population samples. Quechua have the highest recorded allele frequency of T worldwide. Data from Brutsaert et al. (2019) with permission.

The five *EGLN1* SNPs were significantly associated with increased *V*O_2_max in hypoxia (equivalent to an altitude of 4,300 m) after controlling for population stratification (fig. 1*A*). Moreover, SNP alleles associated with high *V*O_2_max are present at highest frequency in Peruvian Quechua compared with 25 diverse lowland populations from the 1000 Genomes Project (fig. 1*B* and *C*). Genotypic effect sizes were large, as comparisons between test subjects that were 5-site homozygotes for the high *V*O_2_max alleles versus those who were alternative homozygotes for the low alleles revealed a statistically significant 13% difference in mean *V*O_2_max (33.97 vs. 30.42 ml·min^−1^·kg^−1^, respectively). The strong association between *EGLN1* variants and *V*O_2_max in Quechua provides physiological context for interpreting the population genomic evidence for positive selection. The enrichment of derived, high *V*O_2_max *EGLN1* alleles in the Quechua population is seemingly consistent with the hypothesis that past selection favored an increased aerobic capacity under conditions of chronic hypoxia or that it favored change in a related, unmeasured phenotype that resulted in increased aerobic capacity as an indirect, carryover effect. Given that the examined SNPs associated with high *V*O_2_max are intronic or are located outside the gene boundaries of *EGLN1*, and given that no coding polymorphisms in the gene were significantly associated with *V*O_2_max, the phenotypic effect of the causal variants must be mediated by changes in gene regulation. The identity of the causal variants, the molecular mechanism by which they exert their effect, and the many intermediary links that connect changes in *EGLN1* regulation with changes in *V*O_2_max remain to be investigated.

### *HIF* Genes, Hemoglobin Concentration, and Aerobic Capacity in Hypoxia

Genotypic associations with aerobic exercise capacity have not yet been documented in Tibetan highlanders, but a recent study demonstrated that *V*O_2_max of Tibetan males at 4,200 m is negatively correlated with hemoglobin (Hb) concentration (Simonson et al. 2015), a phenotype that is associated with allelic variation in HIF genes such as *EPAS1* and *EGLN1* (Beall et al. 2010; Simonson et al. 2010; Yi et al. 2010; Xiang et al. 2013; Peng et al. 2017; Tashi et al. 2017; Yang et al. 2017). In humans and other lowland mammals that ascend to high altitude, Hb concentration increases in a matter of days due to a reduction in blood plasma volume. Subsequently, over the span of >1 week, the elevated Hb concentration is sustained by renal synthesis and release of erythropoietin, a hormone that increases red blood cell production by stimulating the proliferation and differentiation of erythroid precursor cells in the bone marrow (Yoon et al. 2011; Siebenmann et al. 2017). During acclimatization to high altitude, the typical increase in Hb concentration can offset the reduction in arterial O_2_ saturation caused by the reduction in the *P*O_2_ of inspired air, thereby minimizing the reduction in arterial O_2_ content. Thus, if all else is equal, a higher Hb concentration should translate into an increase in convective O_2_ delivery to working muscles and an enhanced aerobic exercise capacity. This is the rationale for blood doping in cycling and other endurance sports. However, at high altitude, the hypoxia-induced increase in Hb concentration does not restore *V*O_2_max to sea level values (Calbet et al. 2002, 2003; Gonzalez and Kuwahira 2018) and it does not necessarily produce net improvements in circulatory O_2_ transport due to antagonistic interactions with interdependent steps in the O_2_-transport pathway.

High-altitude human populations in different parts of the world appear to have evolved different physiological responses to chronic hypoxia and therefore possess different average Hb concentrations at similar altitudes (Beall 2007; Gassmann et al. 2019). Consistent with the acclimatization response of lowland sojourners to high altitude, native Andeans living permanently at altitudes >4,300 m typically exhibit highly elevated blood Hb concentrations (Leon-Velarde et al. 2000). By contrast, Tibetans living at similar altitudes tend to have Hb concentrations that are only slightly higher than values expected for people living at sea level (Beall and Reichsman 1984; Beall and Goldstein 1987; Beall et al. 1998; Wu et al. 2005). At face value, the Tibetan pattern seems counterintuitive, due to the expectation that an increase in arterial O_2_ content should contribute to an enhancement of tissue O_2_ delivery and, hence, improved aerobic performance in hypoxia. However, in spite of their non-elevated Hb concentration, available evidence suggests that Tibetan highlanders have generally superior exercise capacities in hypoxia compared with acclimatized Han Chinese residents living at the same altitude (Sun et al. 1990; Ge et al. 1994; Niu et al. 1995; Chen et al. 1997).

The low (non-elevated) Hb concentration in Tibetans is strongly associated with derived, noncoding variants in and near the *EPAS1* gene that show extreme frequency differences relative to Han Chinese and other lowland reference populations (Beall et al. 2010; Simonson et al. 2010; Yi et al. 2010; Peng et al. 2017; Yang et al. 2017). Although no causal relationship has been established, the fact that non-elevated Hb concentration is associated with derived allelic variants that appear to have increased in frequency due to positive selection suggests that the Tibetan phenotype may be adaptive. This inference is consistent with evidence that excessively elevated Hb concentration is physiologically counterproductive at high altitude (Villafuerte et al. 2004; Storz, Scott et al. 2010; Storz and Scott 2019) and suggests the hypothesis that a blunting of the normal hypoxia-induced increase in Hb concentration evolved as an adaptive mechanism of genetic compensation. One explanation for why the hypoxia-induced increase in Hb concentration may be nonadaptive is that the corresponding increase in blood viscosity compromises cardiac output and microcirculatory blood flow, thereby reducing tissue O_2_ delivery in spite of the increased arterial O_2_ content. An alternative explanation, predicted by results of theoretical models (Wagner 1996), is that an increase in Hb concentration limits the diffusive equilibration of O_2_ between alveolar gas and capillary blood in the lungs (thereby reducing the *P*O_2_ of arterial blood) and between the microcirculatory vessels of muscle and the mitochondria (thereby reducing overall tissue O_2_ extraction). The fact that hypoxia-tolerant highland mammal and bird species typically exhibit Hb concentrations that are within the range of sea-level values for closely related lowland species is also consistent with the idea that hypoxia-induced polycythemia is nonadaptive (Storz, Scott et al. 2010; Storz and Scott 2019).

**Fig. 2.**
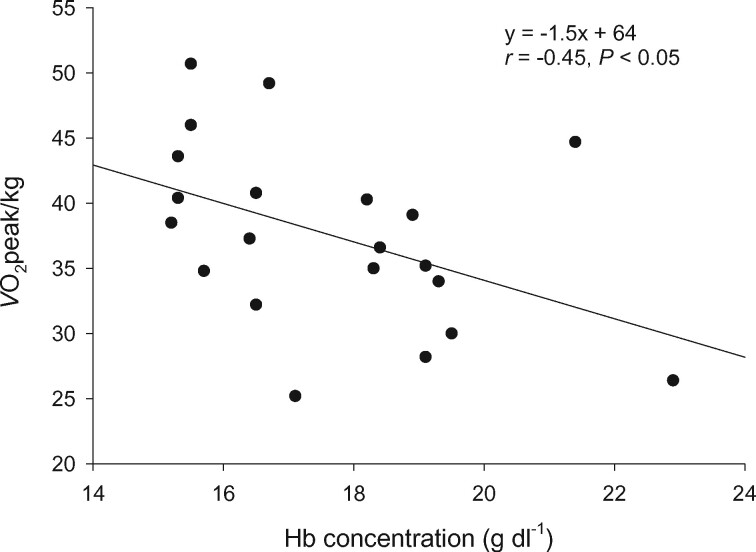
Relationship between Hb concentration and *V*O_2_max in 21 male Tibetan subjects at 4,200 m. From Simonson et al. (2015) with permission.

To assess the physiological consequences of variation in Hb concentration at high altitude, Simonson et al. (2015) investigated the determinants of aerobic exercise capacity by measuring *V*O_2_max and numerous subordinate traits in male Tibetans at 4,200 m. The authors examined each major step in the pathway for O_2_ transport from atmospheric air to the tissue mitochondria: ventilation, pulmonary diffusion capacity (diffusive conductance of O_2_ from the alveoli of the lungs to the pulmonary capillaries), cardiac output, and tissue diffusion capacity (diffusive conductance of O_2_ from tissue capillaries to the mitochondria of metabolizing cells). These linked steps represent conductances that are each expressed as the ratio between O_2_ flow and the O_2_ partial pressure difference across the conductance. Hb concentrations in the test subjects ranged from 15.2 to 22.9 g/dl, consistent with previous studies of Tibetan males at comparable altitudes, and exhibited a significant negative correlation with *V*O_2_max in hypoxia (fig. 2). This study also demonstrated that Hb concentration was negatively associated with cardiac output and O_2_ diffusion capacity of skeletal muscle—the two traits that explained most of the observed variance in *V*O_2_max (Simonson et al. 2015).

### The Challenge of Identifying Phenotypic Targets of Selection

Given the strong evidence for positive selection on *EPAS1* and the association between derived *EPAS1* variants and non-elevated Hb concentration in Tibetans, it has often been implicitly assumed that Hb concentration represents the direct phenotypic target of selection. An alternative hypothesis is that the non-elevated Hb concentration represents a second-order consequence of changes in other traits regulated by HIF-2 that help sustain tissue oxygenation at low inspired *P*O_2_. Hb concentration is regulated by erythropoiesis and water balance via a feedback loop based on renal tissue *P*O_2_ (Donnelly 2003). Thus, attenuation of the hypoxia-induced increase in Hb concentration could be an indirect consequence of changes in any number of steps in the O_2_-transport pathway (ventilation, pulmonary O_2_ diffusion capacity, cardiac output, etc.) that help improve tissue oxygenation, thereby dampening the hypoxic signal that stimulates erythropoiesis and/or plasma volume contraction. Changes in numerous possible respiratory or cardiovascular traits could be mediated by selection on variation in *EPAS1* (Storz, Scott et al. 2010; Petousi et al. 2014; Petousi and Robbins 2014; Simonson et al. 2015; Storz and Cheviron 2016; Storz and Scott 2019).

**Fig. 3.**
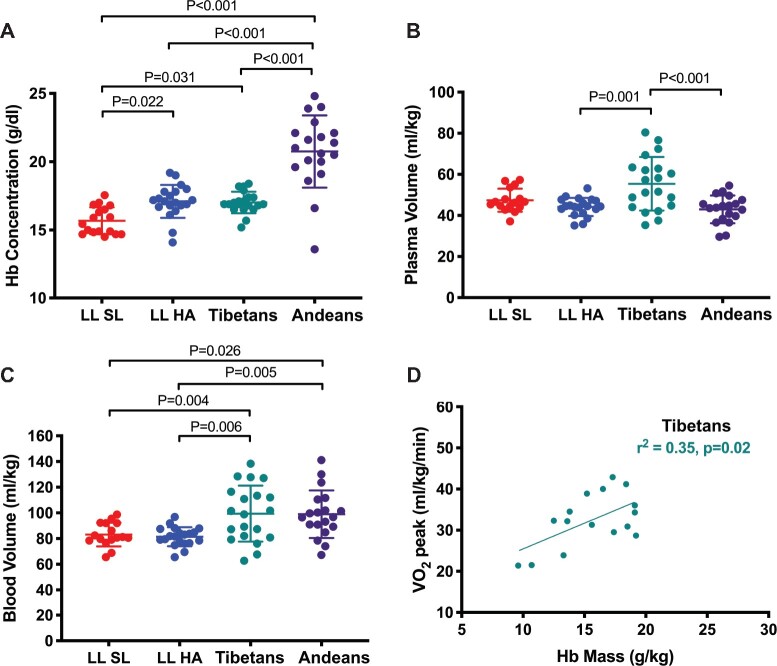
Variation in hematological traits among lowland natives at sea level and acclimatized lowlanders, native Tibetans (Sherpa), and Andeans at high altitude. (*A*) Andeans exhibit an elevated Hb concentration at high altitude relative to acclimatized lowlanders (LL HA) and Tibetans at high altitude. (*B*) Tibetans exhibit a significantly elevated plasma volume compared with acclimatized lowlanders (LL HA) and Andeans at high altitude. (*C*) Due to plasma volume expansion, Tibetans maintain blood volumes that are just as high as those of Andeans, but at a much lower Hb concentration. Consequently, Tibetan highlanders benefit from an augmented blood O_2_ transport capacity while avoiding viscosity-related impairments of cardiac function and microcirculatory blood flow. (*D*) Circulating Hb mass is positively correlated with *V*O_2_max in Tibetans tested at 5,050 m. LL SL, lowland natives tested at sea level; LL HA, lowland natives tested at high-altitude (5,050 m). Modified from Stembridge et al. (2019) with permission.

The non-elevated Hb concentration observed in Tibetan highlanders has traditionally been attributed to a blunted erythropoietic response to chronic hypoxia. However, recent work has demonstrated that Tibetans at high altitude actually have a higher total circulating Hb mass compared with acclimatized lowlanders—indicating that hypoxia-induced erythropoiesis is *not* attenuated—and they also have a considerably higher plasma volume than Andeans and lowlanders tested at similar altitudes (fig. 3*A* and *B*) (Stembridge et al. 2019). Consequently, Tibetans maintain blood volumes that are just as high as those of Andeans, but at a much lower Hb concentration (fig. 3*C*). Tibetans living at high altitude therefore benefit from an increased circulating Hb mass, which augments blood O_2_ transport capacity, and the expanded plasma volume prevents a corresponding increase in Hb concentration, which avoids viscosity-related impairments of cardiac function and microcirculatory blood flow. Accordingly, total Hb mass (but not Hb concentration) was positively correlated with *V*O_2_max in Tibetan subjects tested at 5,050 m (fig. 3*D*). The findings of Stembridge et al. (2019) highlight the importance of considering the functional integration of different components of higher-level performance phenotypes rather than focusing on individual components in isolation.

### *EPAS1* Genotype–Phenotype Associations in High-Altitude Deer Mice

**Fig. 4.**
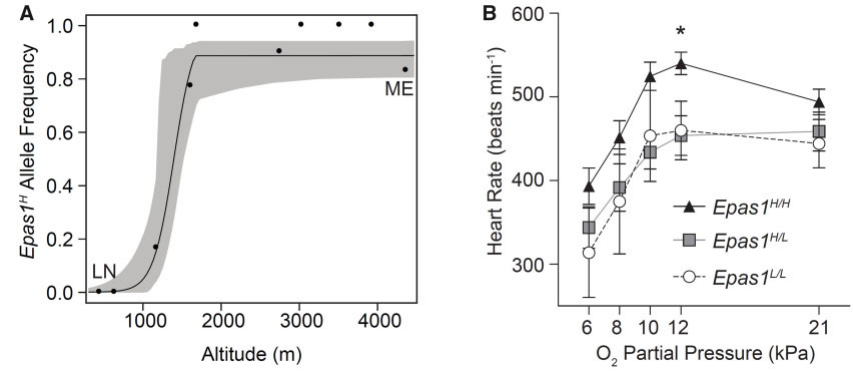
In North American deer mice (*Peromyscus maniculatus*), coding polymorphism in *EPAS1* exhibits a striking pattern of altitudinal variation and contributes to variation in heart rate in hypoxia. (*A*) The derived amino acid variant exhibits a steep altitudinal cline in frequency from the Great Plains to the crest of the Front Range of the Southern Rocky Mountains. LN (Lincoln, NE; 430 m) and ME (summit of Mt. Evans; 4350 m) denote opposite ends of the altitudinal transect. (*B*) When exposed to severe hypoxia (12 kPa O_2_, the *P*O_2_ at the native altitude of the tested mice), high-altitude mice that were homozygous for the highland *EPAS1* variant exhibited significantly higher resting heart rates than mice homozygous for the wild-type allele. From Schweizer et al. (2019) with permission.

Population genomic studies of high-altitude humans and other vertebrates have repeatedly identified *EPAS1* as a candidate gene for hypoxia adaptation (Bigham and Lee 2014; Petousi and Robbins 2014; Simonson 2015; Witt and Huerta-Sanchez 2019; Storz and Cheviron 2021), but functional testing is lacking in all but a few cases and the phenotypic target of selection remains a mystery. An integrated genomic and physiological study of North American deer mice revealed striking evidence for altitude-related selection on *EPAS1* polymorphism but different genotype–phenotype associations than those documented in Tibetan humans (Schweizer et al. 2019). Whereas nucleotide variation is restricted to noncoding sites in *EPAS1* of Tibetan humans (Peng et al. 2011; Hu et al. 2017), a coding polymorphism exhibits the largest altitudinal difference in allele frequency across the *EPAS1* gene of deer mice. This amino acid polymorphism exhibits a steep altitudinal cline in allele frequencies (fig. 4*A*) and genome-wide analyses of nucleotide variation provided strong evidence that the locus-specific pattern of differentiation reflects a history of altitude-related selection.

Using segregating amino acid variation in an alpine population of deer mice living at 4,350 m in the Southern Rockies, Schweizer et al. (2019) tested for associations between *EPAS1* genotype and numerous respiratory, cardiovascular, and metabolic phenotypes. In contrast to the case with Tibetan humans, the highland *EPAS1* variant exhibited no association with Hb concentration (Schweizer et al. 2019). The only measured trait that exhibited a significant association with the derived, highland *EPAS1* variant was an increased resting heart rate under hypoxia (fig. 4*B*). All else being equal, an increase in heart rate increases cardiac output and should therefore increase circulatory O_2_ delivery. During acclimatization to hypoxia, high-altitude deer mice increase cardiac output at *V*O_2_max, a plastic response that makes a significant contribution to aerobic capacity (Tate et al. 2020). Like the case with Hb concentration in Tibetan humans, it is not clear whether the increased heart rate in highland deer mice reflects a direct response to past selection, or whether it represents a secondary consequence of selectively mediated changes in other aspects of O_2_ sensing or O_2_ transport that are regulated by *EPAS1*.

The highland *EPAS1* variant of deer mice was also associated with a downregulation of genes involved in catecholamine biosynthesis in the adrenal gland (Schweizer et al. 2019), a pathway that modulates heart rate and the vasoconstrictive response to hypoxia. Consistent with the observed association in vivo, subsequent experiments revealed that the highland *EPAS1* variant is a loss-of-function mutation that reduces transcriptional activity because it impairs binding of HIF-2α to the transcriptional coactivator CREB-binding protein (Song et al. 2021).

## Female Reproductive Success and the Challenge of Mammalian Pregnancy at High Altitude

Hypoxia-related problems during pregnancy impinge on the most critical period for reproduction and should therefore have an accentuated impact on fitness. Hypoxia contributes to hypertensive disorders (e.g., preeclampsia) and restricted fetal growth (intrauterine growth restriction, IUGR), which greatly increases the risk of stillbirth and infant mortality (Moore 2001; Browne et al. 2015; Niermeyer et al. 2015). Altitude-associated IUGR is typically caused by a slowing of fetal growth during the third trimester of pregnancy, not from a shortening of gestation (Moore 2001). The decline in birthweight with increasing altitude of residence appears to be a universal pattern in all human populations that have been studied, but the birthweight decline in infants born to women of Andean and Tibetan ancestry is roughly half that of infants born to European or Han Chinese women living at similar altitudes (Zamudio et al. 1993; Moore et al. 2001; Julian et al. 2007, 2009; Soria et al. 2013). The reduced susceptibility to IUGR among Andean natives clearly has a genetic basis, as infant birthweights at high-altitude are positively correlated with the fraction of indigenous ancestry of the parents (Julian et al. 2007; Bennett et al. 2008; Soria et al. 2013). These findings are consistent with historical chronicles of life in high Andean settlements during the Spanish conquest of South America, as it was well-documented that mestizo infants born to a Spanish father and indigenous mother had higher rates of survival at high altitude than criollo infants born to Spanish parents (Gonzales 2007). Altitude-associated IUGR also shows a trend in domesticated mammals, as highland breeds or species suffer less fetal growth restriction compared with lowland natives living at the same altitude (Parraguez et al. 2005).

Insights into the genetic and physiological mechanisms that underlie protection from hypertensive disorders of pregnancy and IUGR in high-altitude humans and other mammals could have great medical/veterinary benefits and could greatly enhance our understanding of hypoxia adaptation.

### Genotypic Associations with Components of Female Reproductive Success

Motivated by results of population genomic analyses and a long history of physiological research on Tibetan natives (Beall et al. 2004; Beall 2007, 2014; Gilbert-Kawai et al. 2014), Jeong et al. (2018) used a sample of 1,000 ethnically Tibetan women to test for genome-wide associations with a suite of physiological and reproductive traits. The female study subjects were residents of villages located at altitudes of ∼3,000–4,000 m in the high Himalayan valleys of Nepal. The physiological phenotypes included a set of noninvasive hematological and cardiological measurements and the reproductive phenotypes were based on complete histories of pregnancy outcomes and offspring survival for women at post-reproductive ages. These records represent a rich source of data on lifetime reproductive success in a traditional society where—until recently—women had minimal access to modern medical care and contraceptive birth control (Cho et al. 2017). In addition to testing for genotype–phenotype associations within the set of Tibetan subjects, the authors used the genome-wide polymorphism data to test for evidence of polygenic responses to past selection on the measured phenotypes.

Analysis of genome-wide polymorphism data in the sample of Tibetan women revealed that derived variants at eight SNPs in an intron of *EPAS1* exhibited a strong, negative association with Hb concentration. Data on the reproductive histories of the Tibetan women revealed that lower Hb concentration (measured at post-reproductive ages) is associated with a higher proportion of live births among pregnancies and with lower proportions and lower absolute numbers of still births and miscarriages (Cho et al. 2017; Jeong et al. 2018). This result is consistent with evidence from high-altitude populations that elevated Hb concentration and the associated increase in blood viscosity contribute to IUGR (Gonzales et al. 2009), an effect that may stem from a reduction in uterine artery blood flow and, hence, a reduced O_2_ delivery to the uteroplacental circulation (Zamudio et al. 1995; Moore et al. 2001; Wilson et al. 2007; Julian et al. 2008, 2009; Browne et al. 2015).

Consistent with earlier surveys of genome-wide variation based on different samples of Tibetans (Beall et al. 2010; Simonson et al. 2010; Yi et al. 2010; Peng et al. 2011; Xu et al. 2011; Hu et al. 2017; Yang et al. 2017), Jeong et al. (2018) found that *EPAS1* and *EGLN1* exhibited the strongest signals of positive selection. However, no SNPs in or near either gene exhibited statistically detectable associations with the measured reproductive phenotypes after correcting for multiple tests. This negative result may reflect a lack of statistical power, so it does not necessarily rule out the possibility that the surveyed variants have contributed to a past response to selection on the measured traits. Compared with a reference panel of control SNPs, the *EPAS1* variants associated with low Hb concentration were present at a significantly higher frequency in Tibetans than in lowland reference populations. In Tibetans, this putative signature of polygenic adaptation was driven entirely by a single *EPAS1* SNP where the derived allele associated with low Hb concentration was present at frequencies of 0.75 and 0.01 in Tibetans and Han Chinese, respectively. Based on the estimated genotypic effect sizes for the *EPAS1* SNP alleles and the mean allele frequency difference between Tibetan and Han Chinese populations, Jeong et al. (Jeong et al. 2018) calculated that the *EPAS1* variants explain 52% of the 1.1 g/dl difference in Hb concentration between Tibetan and Han Chinese women in the same age range. However, significant fractions of within- and between-population variation in Hb concentration remain unexplained and—outside of *EPAS1—*no other SNP alleles associated with low Hb concentration in Tibetans exhibited significant frequency differences with lowland populations. On the basis of these results, the authors concluded that Hb concentration may not represent the direct target of selection.

### Association of *PRKAA1* and *EDNRA* Variants with Infant Birth Weight at High Altitude

Using a panel of candidate genes for hypoxia adaptation that were implicated in genome scans of DNA polymorphism in native Andeans, Bigham et al. (2014) tested for genetic associations with altitude-related IUGR and other intermediate phenotypes in a cohort of Bolivian women with native Andean or mixed European ancestry. The authors detected significant associations between maternal genotypes at noncoding SNPs near two genes involved in O_2_ sensing and vascular control, the α-1 catalytic subunit of adenosine monophosphate-activated protein kinase (*PRKAA1*, also known as *AMPK α1*) and endothelin receptor type A (*EDNRA*). *PRKAA1* also exhibited a significant association with a key subordinate trait, uterine artery diameter, an important determinant of uteroplacental blood flow that contributes to protection from altitude-associated IUGR (Zamudio et al. 1995; Julian et al. 2008, 2009; Browne et al. 2015). Finally, the derived *PRKAA1* SNP allele associated with heavier birth weight and larger uterine artery diameter was present at significantly higher frequency in Andeans than in Europeans (0.88 vs. 0.73, respectively), consistent with well-documented differences in altitude-associated IUGR and uteroplacental blood flow in women from these two groups (Wilson et al. 2007; Julian et al. 2009). Interestingly, neither *EPAS1* nor *EGLN1* exhibited significant associations with infant birth weight or uterine artery diameter in the examined cohort of Andean and European women.

Although causal mutations and mechanistic effects have yet to be identified, the associations between specific maternal genotypes and infant birth weight suggest hypotheses about the physiological mechanisms by which high-altitude natives have evolved protection from hypoxia-induced IUGR. The studies of Jeong et al. (2018) and Bigham et al. (2014) focused on associations between maternal genotypes and pregnancy outcomes at high altitude. In future studies of high-altitude humans and other mammals it will also be of interest to investigate the influence of the fetal genotype and parent-of-origin effects (Grant et al. 2020).

## Genetic Experiments Provide Insights into Mechanism and Process in Hypoxia Adaptation

### Modifications of the HIF Pathway

Evidence for positive selection on HIF genes in high-altitude natives has motivated several follow-up studies to examine allelic differences in transcriptional regulation (Peng et al. 2017; Schweizer et al. 2019; Xin et al. 2020), molecular function in relation to HIF signaling (Lorenzo et al. 2014; Song et al. 2014,^ 2021^; Liu et al. 2019) and higher-level physiological phenotypes involved in the response to hypoxia (Petousi et al. 2014; Brutsaert et al. 2019; Schweizer et al. 2019; Song et al. 2020). The product of the *EGLN1* gene (PHD2) binds p23, a chaperone of the HSP90 pathway that promotes folding of client proteins, including HIF-α. The PHD2: p23 interaction recruits PHD2 to the HSP90 pathway, thereby facilitating O_2_-dependent hydroxylation of HIF-α (Arsenault et al. 2016), a posttranslational modification that targets HIF-α for degradation. The putatively adaptive Tibetan PHD2 allele is distinguished from the wildtype (lowland) allele by two amino acid mutations that flank the N-terminal domain responsible for binding p23 and other co-chaperones of the HSP90 pathway (Xiang et al. 2013; Lorenzo et al. 2014; Song et al. 2014, 2020). In vitro experiments revealed that the two mutations impair PHD2: p23 binding (Song et al. 2014), which compromises PHD2-induced hydroxylation of HIF-α. In hypoxia, the reduced rate of hydroxylation promotes the stabilization of HIF-α subunits, thereby facilitating dimerization with HIF-β in the nucleus and the subsequent transcriptional activation of target genes by the HIF-α/β heterodimer. The next question is how this Tibetan-specific modification of HIF signaling affects systemic physiology.

**Fig. 5.**
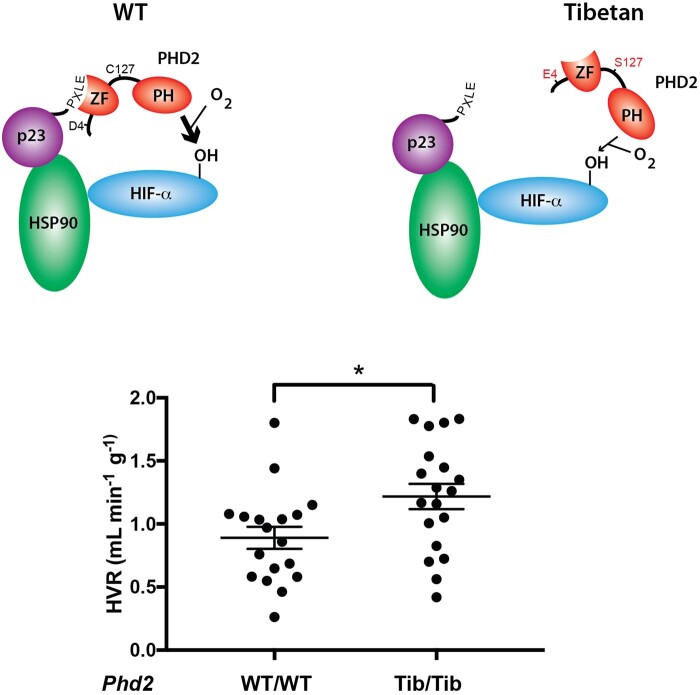
In vivo experiments on knock-in mice reveal that the Tibetan *EGLN1* (PHD2) allele is associated with an enhanced ventilatory response to hypoxia. Relative to mice that carry the wild-type human PHD2 allele, mice that are homozygous for the Tibetan-specific allele exhibit a greater hypoxic ventilatory response (HVR) when exposed to acute hypoxia (12% O_2_/3% CO_2_). The schematic diagram shows that Tibetan-specific mutations at PHD2 sites 4 and 17 impair the binding interaction between PHD2 and p23, a co-chaperone of the HSP90 pathway. Relative to wild-type PHD2, the zinc finger (ZF) binding domain of Tibetan PHD2 binds less readily to the “PXLE” motif of p23. Consequently, Tibetan PHD2 hydroxylates HIF-α less efficiently than the wild-type PHD2 (as indicated by difference in thickness of the arrow connecting PHD2 to HIF-α). Modified from Song et al. (2020) with permission.

In lowland humans, the typical acclimatization response to acute hypoxia involves an increase in ventilation, a response that gradually diminishes with continued exposure (Ivy and Scott 2015; Pamenter and Powell 2016). At high altitude, the breathing pattern of Tibetans is similar to that of newly acclimatized lowlanders (and distinct from that of Andean highlanders) in that they maintain high resting ventilation and an enhanced ventilatory sensitivity to hypoxia at constant arterial CO_2_ concentration (Zhuang et al. 1993; Beall et al. 1997; Brutsaert 2007; Slessarev et al. 2010; Gilbert-Kawai et al. 2014). Experiments on knock-in mice revealed that the double-mutant Tibetan *EGLN1* (PHD2) allele contributed to an augmentation of the hypoxic ventilatory response, recapitulating the Tibetan-specific respiratory phenotype (Song et al. 2020) (fig. 5). These experimental findings suggest that the Tibetan *EGLN1* allele may play a role in mediating genetic assimilation of the ancestral acclimatization response to hypoxia. There is much left to discover regarding interactions between the products of *EGLN1* (PHD2), *EPAS1* (HIF-2α), and other components of the HIF pathway, and the manner in which evolved modifications appear to selectively activate and inhibit different outputs of the pathway in response to hypoxia.

### Modifications of Hb Function, Blood-O_2_ Transport, and Aerobic Capacity in Hypoxia

In addition to population genomic surveys that nominate candidate genes for hypoxia adaptation based on signatures of positive selection, other studies have targeted candidate genes for experimental testing based on known physiological functions. Under conditions of severe hypoxia, theoretical and empirical results suggest that an increased Hb-O_2_ affinity may be adaptive if it helps safeguard arterial O_2_ saturation, and if it is accompanied by an increased tissue O_2_ diffusion capacity so that the augmentation of arterial O_2_ content translates into a corresponding increase in tissue O_2_ extraction (Storz 2016, 2019). Consistent with these predictions, the general trend—far more pronounced in birds than in mammals—is that highland taxa have convergently evolved increased Hb–O_2_ affinities relative to their lowland counterparts (Natarajan, Projecto-Garcia et al. 2015; Natarajan et al. 2016; Storz 2016, 2019; Zhu et al. 2018). Protein-engineering experiments have identified and characterized the specific amino acid replacements responsible for observed changes in Hb function, and have provided detailed insights into molecular mechanisms of biochemical adaptation (Natarajan et al. 2013, 2016, 2018; Projecto-Garcia et al. 2013; Galen et al. 2015; Natarajan, Projecto-Garcia, et al. 2015; Tufts et al. 2015; Kumar et al. 2017; Zhu et al. 2018; Signore et al. 2019; Signore and Storz 2020). In some cases, in vitro experiments that quantified the phenotypic effects of specific mutations have been integrated with population genetic analyses to test for corroborative evidence that the variants in question increased in frequency under the influence of positive selection (Storz and Kelly 2008; Storz et al. 2009, 2012; Storz, Runck et al. 2010; Cheviron, Natarajan et al. 2014; Galen et al. 2015; Natarajan, Projecto-Garcia, et al. 2015).

High-altitude deer mice have evolved a derived increase in Hb-O_2_ affinity relative to lowland conspecifics due to multiple amino acid replacements in duplicated genes that encode the α- and β-chain subunits of the α_2_β_2_ Hb heterotetramer (Storz et al. 2009; Storz, Runck et al. 2010; Natarajan et al. 2013; Natarajan, Hoffmann, et al. 2015; Jensen et al. 2016). Genetic crosses revealed that the evolved increase in Hb-O_2_ affinity contributes to an adaptive enhancement of aerobic capacity at high altitude (Chappell and Snyder 1984; Chappell et al. 1988). To examine the physiological mechanisms by which increases in Hb-O_2_ affinity affect whole-animal performance capacity in hypoxia, Wearing et al. (2020) created F_2_ interpopulation hybrids between highland and lowland deer mice to randomize associations between allelic α- and β-globin variants on an admixed genetic background. They then examined effects of alternative Hb variants on thermogenic *V*O_2_max and subordinate cardiorespiratory and hematological traits in hypoxia. In vivo measurements revealed that the genetically based increase in Hb-O_2_ affinity augments arterial O_2_ saturation in hypoxia (Tate et al. 2017, 2020). However, experimental results and mathematical modeling indicate that the increased arterial O_2_ saturation only translates into an enhancement of hypoxic *V*O_2_max when accompanied by a corresponding increase in tissue O_2_ diffusion capacity (Wearing et al. 2020). It is therefore notable that, in conjunction with the evolved increase in Hb-O_2_ affinity, deer mice native to high altitude have also evolved a skeletal muscle phenotype characterized by enhanced capacities for tissue O_2_ diffusion and O_2_ utilization owing to derived increases in capillary surface density, volume density of total and subsarcolemmal mitochondria, density of oxidative fiber types, and mitochondrial oxidative capacity (Lui et al. 2015; Scott et al. 2015, 2018; Mahalingam et al. 2017, 2020; Tate et al. 2017, 2020; Nikel et al. 2018). These discoveries regarding the determinants of hypoxic *V*O_2_max in deer mice highlight the importance of accounting for the functional integration of focal phenotypes and illustrate how the adaptive value of changes in one trait may be contingent on antecedent changes in other traits.

## Coordinated Evolution of Interdependent Traits

Physiological responses to hypoxia involve coordinated changes in serially integrated traits that exert control over different steps in transport pathways for O_2_ and metabolic substrates (Gonzalez et al. 1993, 1994, 1998; Wagner 1996; Scott and Milsom 2006; Ivy and Scott 2015; McClelland et al. 2017; Tate et al. 2017, 2020; Gonzalez and Kuwahira 2018; McClelland and Scott 2019; Storz and Scott 2019). Consequently, patterns of developmental, functional, and genetic interdependence among such traits may exert a strong influence on the evolution of higher-level performance capacities such as *V*O_2_max.

**Fig. 6.**
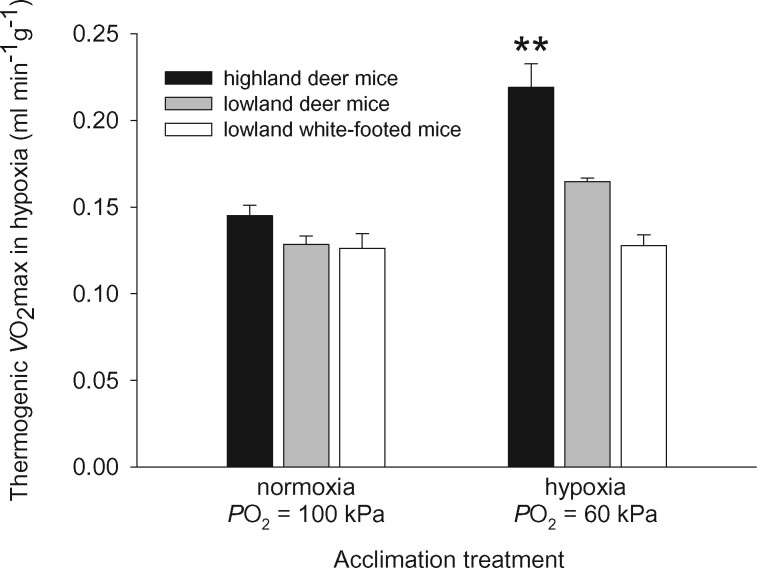
Following acclimation, high-altitude deer mice (*Peromyscus maniculatus*) exhibit a higher thermogenic capacity in hypoxia relative to lowland conspecifics and the exclusively lowland white-footed mice (*P. leucopus*). Thermogenic capacity is measured as cold-induced *V*O_2_max in hypoxia. Data are means ± SEM. **Significant pairwise difference between highland deer mice and both lowland taxa within the same acclimation treatment. ***Data from Tate et al. (2020) with permission.

In hypoxia, high-altitude deer mice have significantly higher aerobic performance capacities than lowland conspecifics, both in terms of exercise-induced and cold-induced *V*O_2_max (Cheviron et al. 2012, 2013; Cheviron, Connaty et al. 2014; Lui et al. 2015; Lau et al. 2017; Tate et al. 2017, 2020). This augmented performance in hypoxia is partly attributable to an interaction between subordinate traits that govern different steps in the O_2_-transport pathway (Tate et al. 2017, 2020). For example, after 6–8 weeks of acclimation to hypoxia (barometric pressure = 60 kPa, *P*O_2_ = 12.5 kPa) at 25 °C, highland mice increase thermogenic *V*O_2_max 1.7-fold, a far more pronounced increase than that observed in lowlanders (fig. 6). The higher thermogenic *V*O_2_max in hypoxia-acclimated highland mice is largely explained by an increase in O_2_ transport to tissues involved in shivering and nonshivering thermogenesis (skeletal muscle and brown adipose tissue, respectively). This enhancement of O_2_-transport capacity in highland mice is attributable to the interaction between a hypoxia-induced increase in cardiac output in conjunction with genetically based increases in arterial O_2_ saturation and tissue O_2_ extraction (Tate et al. 2017, 2020). Evolved changes in these latter two traits in highland mice stem from increases in Hb-O_2_ affinity (Storz et al. 2009; Storz, Runck et al. 2010; Natarajan et al. 2013; Natarajan, Hoffmann et al. 2015; Jensen et al. 2016) and the capillary density and oxidative capacity of skeletal muscle (Lui et al. 2015; Scott et al. 2015, 2018; Mahalingam et al. 2017, 2020; Tate et al. 2017, 2020; Nikel et al. 2018). This example highlights how changes in whole-animal performance capacities may stem from interactions between both plastic and evolved changes in subordinate traits, an important consideration for the design and interpretation of association studies. In physiological studies of deer mice, transcriptomic analyses of O_2_-consuming tissues such as cardiac and skeletal muscle have shed light on mechanisms of plasticity in key phenotypes and have identified changes in regulatory networks that mediate both acclimatization and genetic adaptation to hypoxia (Cheviron et al. 2012; Cheviron, Connaty et al. 2014; Scott et al. 2015; Velotta et al. 2016, 2020; Schweizer et al. 2019).

In the context of high-altitude adaptation, another important question about phenotypic integration concerns the extent to which hypoxia-induced responses in subordinate traits are synergistic or antagonistic with respect to higher-level performance capacities. Studies of hypoxic pulmonary hypertension and other altitude-related maladies in lowland natives indicate that some components of the ancestral acclimatization response to hypoxia are maladaptive. Differences in hypoxia acclimatization between highland and lowland natives suggest that the process of high-altitude adaptation may often involve directional selection on genetically based trait variation that mitigates the deleterious effects of environmentally induced changes. This form of genetic compensation is expected to produce counter-gradient patterns of altitudinal variation in hypoxia-responsive traits such that adaptive phenotypic differentiation between highland and lowland natives is cryptic under field conditions and is only revealed by experimental treatments that control for plasticity (Storz et al. 2010; Storz and Scott 2019, 2021; Storz and Cheviron 2021).

## Future Outlook

Population genomic surveys are useful for generating hypotheses about the genetic basis of high-altitude adaptation, but such data need to be combined with experimental testing to provide insight into functional mechanisms and phenotypic targets of selection. Manipulative physiological experiments are required to determine whether observed genotype–phenotype associations reflect causal effects or indirect consequences of changes in other traits that control interdependent steps in the same pathway. Considerations of phenotypic integration illustrate how observed genotype–phenotype associations can mislead inferences about adaptive mechanisms when the measured phenotype (e.g., Hb concentration) represents a single, labile component of a higher level performance trait (e.g., *V*O_2_max). In Tibetan highlanders, for example, the integrated regulation of erythropoiesis and plasma volume maximizes the benefits of increased Hb mass (augmented arterial O_2_ content) while mitigating associated costs (increased blood viscosity or diffusion limitation). Since reaction norms of hypoxia-responsive traits like Hb concentration do not reveal the underlying pattern of integration with other traits, the causal mechanism underlying the enhancement of circulatory O_2_ transport in hypoxia only came to light once multiple components of trait variation were jointly examined in the context of systemic physiology (Stembridge et al. 2019). Future progress in our understanding of high-altitude adaptation will require integration of genomic data with mechanistic approaches in experimental physiology to dissect the functional, developmental, and genetic interdependence of subordinate traits that contribute to fitness-related performance capacities in hypoxia.
